# Vaccine Dosing Considerations in Product Labels and ACIP Recommendations: A Review

**DOI:** 10.3390/vaccines13070682

**Published:** 2025-06-25

**Authors:** Kunal Saxena, Kate Mevis, Sofia Toso, Elif Alyanak, Natasha Hansen, Aliana Potter, Molly Flannery, Mona Saraiya

**Affiliations:** 1 Outcomes Research, Merck & Co., Inc., Rahway, NJ 07065, USA; kunal.saxena@merck.com; 2 Global Public Policy, Merck & Co., Inc., Rahway, NJ 07065, USA; kate.mevis@merck.com; 3 Avalere Health, Washington, DC 20005, USA; elif.alyanak@avalerehealth.com (E.A.); natasha.hansen@avalerehealth.com (N.H.); aliana.potter@avalerehealth.com (A.P.); 4 Global Regulatory Policy, Merck & Co., Inc., Rahway, NJ 07065, USA; molly.flannery@merck.com; 5 Global Medical Affairs, Merck & Co., Inc., Rahway, NJ 07065, USA; mona.saraiya@merck.com

**Keywords:** vaccine, deviation, ACIP, recommendation, FDA, label, regimen, dosing, schedule, additive, reductive, evidence-based

## Abstract

In the United States, the Food and Drug Administration (FDA) is the regulatory authority with the responsibility to evaluate scientific data included in each vaccine’s prescribing information (e.g., safety, indication(s) for use, and dosing schedule) based on several factors, including safety, quality, potency, and effectiveness in preventing disease to assess benefit/risk prior to approval. After approval, the FDA continues to work with sponsors to ensure safety and effectiveness data in the prescribing information remain current. In conjunction with FDA approval or authorization, the Advisory Committee on Immunization Practices (ACIP) recommends immunization dosing schedules and target populations for use. ACIP recommendations that are adopted by the Centers for Disease Control and Prevention (CDC) Director inform national immunization schedules, which influence immunization access, coverage, and provider behavior. This targeted review aims to explore historical instances when vaccine dosing regimens approved by the FDA differ from those recommended by the ACIP, focusing on the frequency and factors behind these differences to inform future ACIP recommendations. Out of *n* = 78 vaccines assessed, the analysis identified *n* = 5 vaccines with deviations and only one that reduced dosing. Deviations from the FDA label were determined to be a rare occurrence and are most frequently observed to be additive, not reductive.

## 1. Introduction

Vaccines continue to demonstrate widespread societal benefit, playing a pivotal role in ensuring public health and global health [1]. Vaccines have improved health outcomes for millions of individuals, with a recent retrospective analysis finding childhood vaccines saved 154 million lives globally and accounted for 40% of the decline in worldwide infant mortality since 1974 [2]. Additionally, vaccines have provided substantive economic benefits to countries that implement immunization programs. The societal benefit and value of vaccination in the United States have been calculated to be between USD 40 billion and USD 675 billion [3]. For vaccines to be available in the United States, they must be approved by the Food and Drug Administration (FDA) and recommended by the Advisory Committee on Immunization Practices (ACIP). Both review processes require a rigorous evaluation of the safety, efficacy, and potential use of the product, as described below.

In the United States, the FDA licenses vaccines for commercial use. A vaccine manufacturer files a Biologics License Application (BLA) encompassing non-clinical, clinical, safety, and manufacturing data with the FDA. Under unique considerations, a manufacturer may specify other authorizations for use, such as an Emergency Use Authorization (EUA) [4]. The manufacturer seeks approval for the introduction of the vaccine into the commercial market based on the submitted product information, manufacturing information, and pre-clinical and clinical study data [5]. The FDA then evaluates data on safety and effectiveness, as well as product quality and consistency in the vaccine manufacturing processes [6]. The FDA’s evidentiary standards for review and approval are high and include comprehensive and robust assessments of vaccine safety, efficacy, purity, and potency [7,8].

Concurrent with FDA approval, vaccines are reviewed by the ACIP and may be recommended for use following approval or authorization from the FDA. Those recommendations are later adopted by the Centers for Disease Control and Prevention (CDC) Director [9]. This recommendation is a necessary and critical step for vaccines to be integrated into the CDC’s immunization schedule. To develop its recommendations, the ACIP evaluates clinical and safety evidence using the Grading of Recommendations Assessment, Development, and Evaluation (GRADE) framework. With a greater understanding of the benefits and risks associated with vaccination, as assessed via GRADE, the ACIP then reviews additional clinical trial data, real-world evidence, health economics analyses, and implementation science data. These evidence types are assessed using the Evidence to Recommendation (EtR) framework, in which the ACIP evaluates data against specific domains, informing members’ votes on vaccine use [10]. This process allows for input and comments from a variety of stakeholders, such as healthcare providers, public health officials, and the public [11]. Though ACIP recommendations do not override FDA indications, their recommendations have important policy considerations such as implications for insurance coverage, inclusion in the Vaccines for Children Program, and healthcare provider behavior.

Given potential upcoming ACIP discussions on recommendations that may deviate from FDA-approved labels, and the potential public health implications of these recommendations, this review evaluates the historical circumstances in which deviations have occurred and analyzes the unique considerations surrounding each occurrence. This study assessed FDA-approved vaccine labels and their respective ACIP recommendations, using publicly available information.

## 2. Materials and Methods

This study was designed as a targeted review and evaluation of historical vaccine recommendations, within the US specifically, leveraging publicly available data. All ACIP immunization recommendations for 78 FDA-approved vaccines, current as of August 2024, were reviewed. Each vaccine’s product label, pulled from relevant regulatory documentation and filings, was first compared to its respective ACIP-recommended dosing schedule, identified via vaccine use recommendations [9]. The analysis also leveraged any other publicly available FDA and ACIP data current as of August 2024. Based on the findings from this approach, all instances of ACIP recommendations deviating from FDA-approved labels were further reviewed. For the purposes of this research, a “deviation” was defined as an instance in which the ACIP recommends a different dosing schedule for a product than the FDA-approved dosing regimen. Since the ACIP has various dosing schedules under consideration for recommendation development [12], this evaluation focuses on the differences in the dosing regimens. Due to the varying focus on these discussions, ACIP recommendations that deviate from FDA-approved labels as they relate to other areas were not included in this analysis. Other ACIP deviations from approved labels include population deviations, which include recommendations that may modify the population recommended to receive the vaccine as compared to the FDA-approved label. For example, the FDA has approved and licensed respiratory syncytial virus (RSV) vaccines [13,14] for high-risk adults 18 to 59 years, adults aged 60 years and older, and certain adults 50 to 59 years, but the ACIP recommendation [15] narrowed use to adults 75 years and older and certain adults 60 to 74 years.

**Figure 1:**
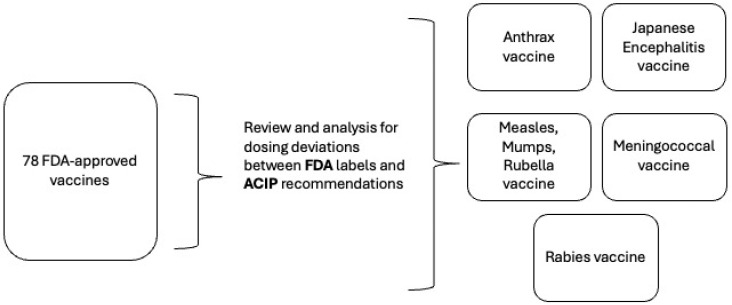
Data identification and analysis process.

FDA-approved dosing regimens were cataloged from each product’s label or package insert, as available within the FDA’s online repository. ACIP recommendation language was collected from CDC-produced immunization schedules, as well as Morbidity and Mortality Weekly Reports (MMWRs) [16,17]. The findings were manually reviewed, validated, and adjudicated as shown in Figure 1.

Limited research exists exploring inconsistencies in regulatory dosing recommendations and actual implementation, particularly among vaccines [18,19,20]. As such, there is no formal definition of a deviation between FDA-approved dosing regimens and recommendations, nor an explicit framework used to best guide this approach.

The ACIP has made other decisions and recommendations around combination products that follow a similar albeit distinct logic, for example, meningococcal vaccines. We chose to highlight the ACIP’s current schedule for meningococcal vaccination, which maintains the same level of antigens as approved by the FDA while changing the explicit vaccine doses given the incorporation of the MenABCWY vaccine. This allowed us to underscore potential further discussions around combination products that adjust the dosing regimen yet maintain adequate protection as originally approved by the FDA.

## 3. Results

This analysis found that among all 78 FDA-approved vaccines, five ACIP dosing recommendations deviated from FDA-approved labels. These five recommendations are for anthrax; Japanese encephalitis (JE); measles, mumps, and rubella (MMR); meningococcal; and rabies vaccines and are categorized based on the type of dosing deviation that is assessed in Table 1.

### 3.1. Anthrax

The ACIP’s anthrax dosing recommendation deviates from the respective labeled product antigen dosing information by increasing the dosing regimen for post-exposure prophylaxis (PEP). There are two FDA-approved products for anthrax PEP: Cyfendus^®^ [22] is indicated for two doses, while Biothrax^®^ [23] is indicated for three doses. The ACIP’s recommendation [24] for three doses aligns with the FDA-approved dosing regimen for Biothrax^®^ but not with the FDA-approved dosing regimen for Cyfendus^®^. This recommendation deviates from the label because the ACIP reviewed Biothrax^®^ prior to the FDA approval of Cyfendus^®^ and has not reconvened to deliberate on anthrax vaccination since its initial recommendation. As such, the recommendation as it stands is an additive dosing recommendation that allows for consistency, regardless of the product used.

### 3.2. Japanese Encephalitis

The ACIP’s JE recommendation deviates from the respective FDA-approved dosing regimen by strengthening language around a booster dose. The FDA-approved product label [25] for JE states “a booster dose may be given”, while the recommendation states, “a booster dose should be given” [26]. This recommendation shifted the regimen from potential shared clinical decision-making (SCDM) to a routine recommendation for boosting, resulting in an additive dosing recommendation [26].

### 3.3. Measles, Mumps, and Rubella

The ACIP’s MMR dosing recommendation deviates from the respective FDA-approved dosing regimen by recommending an additional third dose for specific populations in outbreaks. While the FDA-approved product label for MMR vaccines indicates a two-dose series [27,28,29], the ACIP recommendation [30] includes further language specifying, “a third dose for persons previously vaccinated with two doses of a mumps-virus-containing vaccine who are identified by public health authorities as being part of a group or population at increased risk for acquiring mumps because of an outbreak.” After finding the vaccine safe and effective, the ACIP recommended an additional dose of MMR vaccination in these outbreak settings. The ACIP determined that, in the event of an outbreak, the benefit of added protection through the administration of a third dose of MMR vaccine outweighs the low risk for vaccine-associated adverse events, leading to an additive recommendation [30].

### 3.4. Meningococcal

The ACIP’s recommendations for meningococcal vaccination [31] deviate from the respective FDA-approved product dosing information [32,33,34,35,36,37] by presenting alternative dosing regimens but ultimately maintaining the same level of protection. The introduction of a new pentavalent meningococcal vaccine (MenABCWY) in 2023 illustrates an example of an ACIP recommendation that deviates from the FDA-approved dosing regimen but maintains the protection that individuals receive against each of the included serotypes and strains. Historically, prior to the introduction of MenABCWY vaccines, the index meningococcal schedule included recommendations for two doses of the quadrivalent MenACWY vaccine (either available/licensed vaccines, interchangeable for doses) and a SCDM recommendation for two doses of the monovalent MenB vaccine (non-interchangeable) [31]. With the approval of the pentavalent vaccine, which consolidated all previously recommended serotypes (MenABCWY) into one vaccine, the ACIP reassessed the existing schedule. Following its review, the ACIP chose to maintain the existing schedule, supplementing it with a recommendation that the pentavalent MenABCWY vaccine could be used as an option during visits where individuals were recommended both one dose of MenACWY and one dose of MenB [38]. As a result, when a provider administers the full meningococcal series, they may either follow a scenario to (1) provide two doses of quadrivalent MenACWY vaccine and two doses of monovalent MenB vaccine or (2) provide one dose of MenACWY, one dose of MenABCWY, and one dose of MenB.

The FDA-approved label for each of these products (MenACWY, MenB, and MenABCWY) indicates the use of two doses for demonstrated protection. The ACIP’s decision to deviate from the label was driven in part by concerns about increased MenB antigen exposure, specifically among individuals at younger ages who historically were not recommended to routinely receive MenB vaccines. This included concerns regarding reactogenicity, a low burden of disease, and limitations to protection [39].

### 3.5. Rabies

The ACIP recommendation for rabies vaccination deviates from the respective label by reducing the antigen dosing regimen while still maintaining the prime plus boost paradigm. The prime plus boost paradigm refers to the enhanced immune response gained from an additional dose of vaccine [40]. In the use of the rabies vaccine for pre-exposure prophylaxis (PrEP), the FDA-approved label lists a three-dose series [41,42], but in its recommendation development process, the ACIP reduced the regimen to a two-dose series [43]. Similarly, for PEP, the FDA-approved label indicates a five-dose series, but the ACIP recommends a four-dose series [44]. To supplement its decision to remove or not recommend the fifth dose of rabies vaccine, the ACIP’s PrEP and PEP recommendations include a booster dose and guidance for extensive antibody titer monitoring. Specifically, the ACIP recommends a booster dose for individuals in certain risk categories if antibody titers fall below 0.5 IU/mL [45].

ACIP reduced the number of doses used for the PrEP and PEP of rabies vaccines for various reasons, including the high out-of-pocket (OOP) cost of the vaccine and non-compliance with titer checks [43]. For PEP, the MMWR cited studies indicating that a four-dose schedule in combination with rabies immunoglobulin elicited adequate immune responses, whereas a fifth dose did not create a more favorable outcome [44]. The GRADE evidence for PrEP indicated that two doses provided adequate protection through a three-year timepoint [46]. The ACIP justified the recommendation based on several barriers to vaccination including high out-of-pocket costs, confusion about which category of vaccination individuals fell into, and noncompliance with titer checks [43].

## 4. Discussion

While some research has previously been conducted on the topic of vaccine recommendation deviations, this paper fills a research gap by providing a more current analysis, focusing on dosing deviations, and addressing a broader set of healthcare system implications specifically within the US [47,48]. In an evaluation of 78 FDA-approved vaccines, five cases were identified in which the antigen dosing on the FDA-approved label and in the ACIP recommendation differed. The ACIP recommendation for rabies immunization is the only example where an ACIP recommendation deviates from FDA-approved labels by reducing the dosing regimen without allowing for an alternative regimen. However, the rabies vaccine deviation is an imperfect case study to showcase the differences between an FDA-approved label and an ACIP recommendation. This case study is not broadly generalizable when compared to other efforts to deviate in dosing, as recommendations for rabies vaccination are distinctively not for routine use but rather in PrEP and PEP for an extremely severe and fatal disease. Additionally, a very limited number of individuals require this vaccine annually, allowing for easier monitoring, surveillance, and follow-up [49].

Apart from the rabies vaccine case study identified in this analysis, the ACIP has historically followed the proven clinical threshold for dosing recommendations to support prevention as dictated by FDA-approved labels, which are informed by large-scale, methodologically rigorous clinical trials submitted as part of the manufacturer’s evidence of safety and effectiveness. Further, during the COVID-19 pandemic, the ACIP had originally chosen to not recommend a booster dose for high-risk groups, but this decision was later amended by the CDC Director’s final recommendation to include the booster dose for specific adults at high risk due to “occupational and institutional exposures”, aligning the dosing regimen with the FDA-approved Emergency Use Authorization at the time [50]. Similarly, in 2014, the ACIP Pneumococcal Workgroup (WG) considered reducing the pneumococcal dosing schedule for infants from a four-dose regimen to a three-dose regimen. The WG was considering this change because of evidence from other countries using different dosing schedules. Due to limited evidence and the potential far-reaching consequences of such a change, the WG decided not to move forward with considering reduced dosing [51,52].

Not only do these examples demonstrate that the ACIP and CDC frequently follow evidence-based standards, but the overwhelming precedent for these ACIP recommendations ensures that providers adhere to the proper dosage and that payers cover dosage amounts in line with FDA decisions.

When the ACIP’s recommendation deviates from the FDA-approved label, changes that differ from that basis of regulatory approval, particularly as it relates to dosing, have a potential cascading impact by conferring a reduced level of effectiveness and/or prompting other implementation concerns. Though this analysis demonstrates that deviations from FDA-approved dosing regimens for vaccination are rare and specific, further deviations could result in downstream implications, including the following:

- Secondary Prevention: Changes to dosing regimens may place an additional need on secondary prevention strategies, like screening and testing. Decisions related to these secondary prevention tools are often made by different recommending bodies [53], complicating efforts to ensure the systems of prevention in place are complementary.
- Disease Surveillance: The main dosing recommendation that reduces the level of protection as compared to the label is for pre- and post-exposure rabies vaccinations [44], which involves a generally limited population that allows for surveillance and tracking. Recommendations for vaccines meant for larger cohorts have additional and different complexities in tracking disease prevention effectiveness; these challenges are likely greater for the long-term monitoring of large cohorts of populations who receive protection through a routine recommendation.
- Vaccine Hesitancy: It is possible that deviated dosing could raise concerns among stakeholders and misperceptions about why there is a conflict between government agencies regarding the appropriate dosing for a vaccine. Additionally, patients may question why the ACIP initially recommended the additional dose series if a reduced dose series is adequate to be effective, calling into question the standard of evidence that is required for the ACIP compared to that for the FDA.
- Parent and Provider Confusion: Uninformed providers may be confused regarding the correct number of doses to be administered, which could lead to out-of-pocket costs for patients, and parents may question the driver of the dose reduction. Additionally, further updates to the dosing schedule for vaccines could raise concerns among parents and providers. Harmonizing the guidance across these agencies and advisory committees may help to mitigate confusion among these stakeholders and may allow for simplified decision-making (e.g., material development for providers) and more consistent public health guidance.

The few instances in which a dosing deviation between the label and recommendation have occurred suggest that the ACIP and CDC take into consideration these and other challenges, which may be associated with deviated dosing recommendations throughout the process of their comprehensive EtR review, including specific domains related to feasibility, acceptability, and resource use (e.g., cost-effectiveness). However, as the role of immunization is most often primary prevention against disease [54], aligning ACIP recommendations with dosing information in the FDA-approved label emphasizes that the evidence-based dosing and schedule have been determined to be potent and effective in disease prevention as approved by the FDA.

## 5. Conclusions

This evaluation found that dosing deviations between ACIP recommendations and FDA-approved labels are rare and specific to each vaccine and disease area. These deviations have focused on ensuring effectiveness while maintaining safety.

The systematic evidence review and appraisal processes that inform ACIP recommendation development are heavily influenced by the FDA’s reputation for high standards and comprehensive evaluations that a product’s dosing is established as safe and effective; deviations from these approved uses can result in uncertainty and implementation challenges for important immunizations. Aligning the ACIP recommendation with the FDA-approved label, as is the case for most products, follows common practice to create evidence-based recommendations for safe vaccine use and ensures vaccines meet the high bar of safety and efficacy that forms the basis of regulatory approval.

## Figures and Tables

**Table 1:** Vaccines with ACIP recommendations deviating from FDA dosing regimen.

Vaccine	Type of Deviation
Anthrax	Additive Dosing: Recommendations call for an additional dose beyond the product’s FDA-approved dosing regimen to offer supplemental protection to certain populations in specific situations (e.g., outbreaks).
Japanese Encephalitis	
Measles, Mumps, Rubella	
Meningococcal	Maintains Dosing: Recommendation maintains the protective dosing amounts within the overall schedule but may be administered differently than product labeling because of the unique combinations of single- and multi-serotype vaccines.
Rabies	Reductive Dosing: Recommendation removes a dose from what is otherwise described on the label, recommending four doses instead of five. This dosing schedule retains the prime plus boost paradigm, which is when an additional dose is needed to “remind” the immune system of a disease [21].

## Data Availability

The data were derived from the following resources available in the public domain: CDC.gov and FDA.gov.

## Abbreviations

The following abbreviations are used in this manuscript:

FDA	Food and Drug Administration
ACIP	Advisory Committee on Immunization Practices
CDC	Centers for Disease Control and Prevention
BLA	Biologics License Application
EUA	Emergency Use Authorization
GRADE	Grading of Recommendations, Assessment, Development and Evaluation
EtR	Evidence to Recommendation
RSV	Respiratory Syncytial Virus
MMWR	Morbidity and Mortality Weekly Report
JE	Japanese Encephalitis
MMR	Measles, Mumps, and Rubella
PEP	Post-Exposure Prophylaxis
SCDM	Shared Clinical Decision-Making
PrEP	Pre-Exposure Prophylaxis
OOP	Out-of-Pocket
WG	Workgroup
